# Activation and pro-inflammatory cytokine production by unswitched memory B cells during SARS-CoV-2 infection

**DOI:** 10.3389/fimmu.2023.1213344

**Published:** 2023-08-10

**Authors:** Moriah J. Castleman, Adriana Luna Santos, Kelsey E. Lesteberg, James P. Maloney, William J. Janssen, Kara J. Mould, J. David Beckham, Roberta Pelanda, Raul M. Torres

**Affiliations:** ^1^ Department of Immunology and Microbiology, University of Colorado School of Medicine, Aurora, CO, United States; ^2^ Department of Medicine, Division of Infectious Disease, University of Colorado School of Medicine, Aurora, CO, United States; ^3^ Department of Medicine, Division of Pulmonary Sciences and Critical Care Medicine, University of Colorado School of Medicine, Aurora, CO, United States; ^4^ Department of Medicine, National Jewish Health, Denver, CO, United States; ^5^ Department of Medicine, University of Colorado, Aurora, CO, United States; ^6^ Rocky Mountain Regional VA, Medical Center, Aurora, CO, United States

**Keywords:** unswitched memory, switched memory, B cells, COVID - 19, SARS-CoV- 2, human, TNF-a, autoantibodies

## Abstract

Memory B cells are comprised of unswitched (CD27+IgD+) and switched (CD27+IgD-) subsets. The origin and function of unswitched human memory B cells are debated in the literature, whereas switched memory B cells are primed to respond to recurrent infection. Unswitched memory B cells have been described to be reduced in frequency with severe SARS-CoV2 infection and here we characterize their activation status, BCR functionality, and contribution to virally-induced cytokine production. Analyses of whole blood from healthy individuals, people immunized against SARS-CoV2, and those who have had mild and severe SARS-CoV2 infection, confirm a reduction in the frequency of unswitched memory B cells during severe SARS-CoV2 infection and demonstrate this reduction is associated with increased levels of systemic TNFα. We further document how severe viral infection is associated with an increased frequency of ‘IgD+’ only memory B cells that correlate with increased IgG autoantibody levels. Unswitched and switched memory B cells from severe SARS-CoV2 infection displayed evidence of heightened activation with a concomitant reduction in the expression of the inhibitory receptor CD72. Functionally, both populations of memory B cells from severe SARS-COV2 infection harbored a signaling-competent BCR that displayed enhanced BCR signaling activity in the unswitched population. Finally, we demonstrate that B cells from mild SARS-CoV2 infection are poised to secrete pro-inflammatory cytokines IL-6 and TNFα. Importantly, unswitched memory B cells were a major producer of IL-6 and switched memory B cells were a major producer of TNFα in response to viral TLR ligands. Together these data indicate that B cells contribute to the inflammatory milieu during viral infection.

## Introduction

CD27 and IgD expression identify four major B cell subsets within the peripheral blood of humans. These are the unswitched memory B cells that express both surface proteins (CD27+IgD+), switched memory B cells that express CD27 but not IgD (CD27+IgD–), naive/transitional B cells that are newly recruited from the bone marrow and express IgD but not the memory marker CD27 (CD27-IgD+), and double negative B cells, which are comprised of heterogenous subsets that do not express either marker (CD27-IgD-) (1). The characterization of phenotype and functional status of these various human B cell subsets has begun to highlight the unique function of each population in health and disease.

Switched memory cells are typically considered classical memory B cells that originate in germinal centers, are long-lived and antigen experienced, and that can gain a considerable number of mutations in their immunoglobulin variable genes through affinity maturation. Switched memory B cells are primed to respond to a recurrent infection by proliferation and differentiation into plasma cells or re-entry into germinal centers (2). This population includes B cells that have class switched their immunoglobulin isotype from the default IgM and IgD to either IgG, IgA, or IgE. Additionally, there is a small subset of ‘IgM only’ cells in the switched memory population (CD27+IgD-IgG-IgA-IgE-), which are thought to be an early germinal center emigrant subset that has lost expression of IgD but has acquired a low level of immunoglobulin mutations in IgM (3, 4). Given the clear role of switched memory B cells, particularly the IgG and IgA isotype classes in response to infection (3), this population is more widely studied than unswitched memory B cells.

Unswitched memory cells are a non-classical and more recently appreciated type of memory B cell whose origin in humoral immunity remains controversial (2, 5). Unswitched memory (CD27+IgD+) B cells comprise two populations including IgM-expressing B cells (CD27+ IgD+IgM+) and the population that has lost IgM expression (CD27+ IgD+IgM-) thus becoming ‘IgD+ -only’ B cells, which are cells that have truly class-switched at the immunoglobulin locus through DNA recombination (1, 6–8). Unswitched memory B cells have also been referred to in the literature as ‘marginal zone-like’ or ‘non-switched memory’ B cells (2, 5). The term ‘marginal zone B cells’ originated due to similarities between human CD27+IgD+ B cells with marginal zone B cells found in the spleen of mice that reside on the periphery of B cell follicles and have a unique phenotype (IgM^hi^ IgD^lo^ CD21+ CD23- CD1+) (9). Furthermore, asplenic individuals have reduced frequencies of IgM-memory B cells and display a high incidence of infection by encapsulated bacteria (10), consistent with the ascribed function of mouse marginal zone B cells in defending against encapsulated bacteria (11). Thus, some consider unswitched memory B cells in the periphery blood of humans to be the equivalent to marginal zone B cells in mice, however, key differences between these cell types have been reported (5). For simplicity, (and despite the possibility of a marginal zone origin) in this manuscript we have termed all circulating cells identified by CD27+IgD+ to be unswitched memory B cells. Examination of the BCR repertoire revealed that unswitched memory cells have less clonal expansion than switched memory B cells and differences in amino acid usage, intimating these populations develop through differing pathways (12) and thus could serve unique functions in health and disease. Whereas the role of switched memory B cells in humoral immunity has been described (3, 13), the role of unswitched memory B cells in response to vaccination and viral infection is still unclear.

There have been reports of reductions in the frequency of the unswitched memory B cell population in various autoimmune diseases, including Sjögren’s syndrome (SS) (14), systemic lupus erythematosus (SLE) (15, 16), and rheumatoid arthritis (RA) (17), and in SLE and RA patients with highly active disease a larger reduction was noted (14, 16, 17). Importantly, the frequency of unswitched memory B cells reverted to normal levels upon disease remission post-treatment both in SLE with steroids (15) and in RA with anti-TNFα treatment (17), proposing that disease-associated inflammation and in particular levels of TNFα may promote loss of unswitched memory B cells. The contribution of unswitched memory B cells to autoimmune disease development remains unclear. A negative correlation between levels of autoantibodies and frequency of unswitched memory B cells has indicated this subset may be protective against autoimmunity. Specifically, SLE patients with higher levels of autoreactive antibodies have been found to harbor fewer unswitched memory B cells (15, 16). Similarly, a negative correlation between levels of anti-SSa autoantibodies and the frequency of unswitched memory B cells was reported in SS (14). Additionally, the IgD+-only subset of unswitched memory B cells is enriched in highly autoreactive and polyreactive BCRs (7) and has undergone extensive receptor editing in healthy controls (8), indicating at least this population may contribute to autoimmunity.

There are limited reports on the functional capacity of unswitched memory B cells both in human health and disease. Unswitched memory B cells were reported to be functionally impaired in patients with SLE or RA as measured by reduced secretion of IgM after *in vitro* stimulation with anti-CD40 (mimicking T cell help) and TLR9L (CpG) compared to unswitched memory B cells from healthy controls (15, 17). Comparison of unswitched to IgG-expressing switched memory B cells from healthy controls revealed that unswitched memory B cells produce less immunoglobulin in response to antigen stimulation (18). While the dominant and most well-studied function of B cells is antibody production, an under-appreciated function of B cells is cytokine production (19, 20). Human memory B cells (including unswitched and switched) were reported to produce higher levels of pro-inflammatory cytokines IL-1β, IL-6, and TNFα than naive B cells in response to stimulation *via* TLR ligands independent of antigen stimulation (21), although the contribution of unswitched memory B cells to cytokine secretion was undetermined. RNA-sequencing revealed that *IL6* gene expression was two-fold higher in unswitched memory B cells compared to switched memory B cells in healthy controls, suggesting unswitched memory B cells are more poised to produce this pro-inflammatory cytokine than switched memory B cells (9). In the context of SLE, unswitched memory B cells had reduced IL-10 secretion as compared to healthy controls (15). These studies support the possibility that unswitched memory B cells have differential functions compared to switched memory B cells and, at least in autoimmune disease, there is a defect in function in the unswitched memory compartment. It also suggests that the total unswitched memory B cell population may respond to TLR ligands in an innate manner, independent of antigen stimulation. However, the function of unswitched memory B cells in individuals with viral infection, particularly in the context of cytokine production, has not been reported.

Multiple groups have reported that severe infection with Severe acute respiratory syndrome coronavirus 2 (SARS-CoV-2) virus, the causative agent of the COVID-19 pandemic induces a reduction in the frequency of circulating unswitched memory B cells (22–27). However, examination of circulating unswitched memory B cells two months post-mild SARS-CoV-2 infection did not demonstrate any differences in the frequency of these cells compared to healthy controls (28, 29), suggesting that the severity of the disease may impact a reduction in this population. Supporting this concept, the frequency of circulating unswitched memory B cells negatively correlated with systemic levels of CRP and disease severity (22). Furthermore, the frequency of unswitched memory B cells clustered with the clinical outcome of discharge from the hospital (25), demonstrating this population may be protected from viral infection. Despite these reports, the phenotype and function of unswitched memory B cells during SARS-CoV-2 infection remain poorly characterized during viral infection or vaccination.

In this study we collected PMBCs and plasma from healthy controls, people immunized against SARS-CoV-2, or those with mild or severe SARS-CoV-2 infection. Total memory B cells (independent of antigen specify) were examined in these four cohorts for phenotypical and functional characteristics and comparisons between unswitched and switched B cells were explored. We confirm a reduction in the frequency of the unswitched memory B cell population with a severe viral infection and further characterized the activation and loss of inhibition in this population. Importantly, we report an expansion of the autoreactive IgD+only subset in severe SARS-CoV-2 infection, which correlated with increased levels of autoreactive antibodies. We also demonstrate an enhanced BCR signaling ability by the unswitched memory B cell population and a heightened production of pro-inflammatory cytokines by these cells in response to viral TLR ligands. Together these data highlight the novel roles of the unswitched memory B cell population during viral infection.

## Materials and methods

### Human peripheral blood mononuclear cells

Samples used in this study for the severe SARS-CoV-2 cohort were from individuals hospitalized in the ICU St. Joseph’s Hospital (SJH Denver) or at the University of Colorado Hospital (UCH). An approved waiver of consent (UCH) or a legally authorized representative (SJH) provided informed consent to donate whole blood. Individuals who donated blood had a nasal swab to confirm the presence of the virus by polymerase chain reaction (PCR), were at least 18 years old, and mechanically ventilated for acute respiratory distress syndrome. The exclusion criteria for this study included individuals with solid organ or bone marrow transplants, hemoptysis, chronic lung disease, pregnancy, increased risk for bleeding, or immunosuppressed. Whole blood was collected from central venous catheters in sodium citrate collection tubes and processed per the manufacturer’s instructions (BD Biosciences, San Jose, CA), with plasma and PBMCs stored as previously described (30, 31).

The mild SARS-CoV-2 infection cohort samples were included in this study if they were confirmed to have the virus by nasal swab PCR or the presence of anti-SARS-CoV2 antibodies, did not require hospitalization for infection, and were able to donate whole blood in the convalescent stage. These samples were collected before vaccination was available to the general public. The samples used in this study for the immunized cohort were from individuals vaccinated against SARS-CoV-2 by Pfizer BNT162b2-mRNA or Moderna mRNA-1273 who were able to provide dates of their primary inoculation and booster. Informed consent was provided by these individuals. Whole blood was drawn by the Colorado Clinical and Translation Sciences Institute (CCTSI). PBMCs and plasma were collected and stored as previously described (30, 31).

Samples from healthy controls were obtained from Vitalant Blood Center (Denver, CO). PBMCs were isolated from plateletpheresis leukoreduction filter (LRS chambers) as previously described (30, 31). Since LRS chambers do not allow for the acquisition of plasma, in this study when plasma was examined, the immunized individuals were used as a comparator to SARS-CoV-2 individuals.

The Colorado Multiple Institutional Review Board (COMIRB) approved the use of human samples and this study, which was performed under the Declaration of Helsinki.

### Frequency and phenotyping of memory B cells *ex vivo* by flow cytometry

To characterize the frequency and phenotype of memory B cells, PBMCs were thawed as previously described (30, 31). PBMCs were stained on ice for 20min in 1X PBS with antibodies (clone): CD3 (OKT3), CD27 (M-T271), CD19 (SJ25C1), IgD (IA6-2), CD69 (FN50), CD86 (IT2.2), CD21 (Bu32), CD22 (HIB22), CD72 (REA231), IgM (MHM-88), and IgG (G18-145) with Live/Dead Blue for UV viability dye (Invitrogen, Eugene, OR). After surface stain, PBMCs were washed and fixed with 4% PFA (Fisher, Fair Lawn, NJ). To run on the flow cytometer, cells were resuspended in 1% BSA with 0.05% Sodium Azide in 1X PBS.

The spectral Cytek Aurora was utilized to acquire all flow cytometry data and data were analyzed using FlowJo software (v 10.8.1). Single color reference controls were generated using PBMCs from healthy humans. When necessary, Ultra Comp eBeads (Invitrogen) were used. Gating through lymphocytes, singlets, live cells, and CD3- cells, unswitched memory B cells were identified as CD19+ CD27+ IgD+. The IgD+only subset within unswitched memory B cells was identified by then gating on IgM negative cells. Gating through lymphocytes, singlets, live cells, then CD3- cells, switched memory B cells were gated as CD19+ CD27+ IgD-. The frequency of unswitched or switched memory B cells were enumerated out of the combined memory population (B cells that are CD27+) or out of total B cells (CD19+).

### Assessment of BCR signaling by phospho-flow cytometry

To evaluate signaling downstream of the BCR in memory B cells, PBMCs were thawed then incubated in warm serum-free RPMI Medium 1640 for 45 min at 37˚C with 5% CO2 to reduce basal phosphorylation levels while in the presence of the following antibodies (clone): CD3 (OKT3), CD27 (M-T271), and CD19 (SJ25C1) with Live/Dead Blue for UV viability dye. PBMCs were resuspended in warm RPMI with 5% FBS and stimulated with either 10ug/mL Rabbit anti-human IgG (H+L) F(ab’)2 (Southern Biotech, Birmingham, AL) or positive control 75μM pervanadate for 5 minutes in a 37˚C water bath. After stimulation, PBMCs were BD Cytofix/Cytoperm for 20 minutes on ice and washed in 1X Perm/Wash (BD). The following antibodies (clone): pSYK Y348 (I120-722), IgD (IA6-2), and pPLCγ2 Y759 (K86-689.37) were used for intracellular staining for 30 min on ice in 1X Perm/Wash before data acquisition.

### Quantification of secreted cytokines by purified B cells upon *in vitro* stimulation

PBMCs were thawed as described above and total B cells were enriched using the Mojo Human anti-CD3 selection kit (Biolegend, San Diego, CA) with the addition of anti-CD16-biotin (clone 3G8) and the Easy Sep magnet (Stem Cell Technologies, Cambridge, MA) to deplete non-B cells. Enriched B cells were then incubated on ice for 20min with the following antibodies CD3 (OKT3) and CD19 (SJ25C1) in the presence of Zombie green viability dye (Biolegend), washed in 1X PBS and resuspended in sorting buffer comprised of 1X PBS with 1mM EDTA, 25mM HEPES, and 1% BSA then sorted on the BD Aria Fusion Cell Sorter at the University of Colorado Allergy and Clinical Immunology Flow Cytometry Facility. Purified B cells were plated in 96 well tissue culture plates in RPMI 1640 media with 10% FBS, 1X Glutamax, and 1X Penicillin/streptomycin (Gibco) for 18 hours at 37˚C with 5% CO2 in the presence of 50ng/mL PMA (Sigma) with 1ug/mL Ionomycin (Sigma) or 10ug/mL anti-Ig (H+L) F(ab)’2 (Southern Biotech) and 100ng/mL CD40L (Biolegend) with either 1ug/mL TL3RL (Poly I:C), 1ug/mL TLR7/8L (R848), or 2.5ug/mL TLR9L (CpG) (all from InvivoGen, San Diego, CA) as indicated in the figure legends. The supernatant was collected after incubation and cytokines were enumerated using the U-PLEX Assay according to the manufacturer’s instructions (Meso Scale Discovery, Rockville, MD). The QuickPlex SQ 120 Instrument was used the read the Mesoscale plate (Meso Scale Discovery).

### Intracellular cytokine staining of human memory B cells upon *in vitro* stimulation

To determine whether memory B cells were capable of producing IL-6 and TNFα cytokines, PBMCs were thawed, incubated for 15 min on ice with antibodies IgD (IA6-2) and IgM (MHM-88), washed by centrifugation 1,500 rpm for 5min in 1X PBS then incubated for 4 hours at 37˚C with 5% CO2 in RPMI 1640 media with 10% FBS, 1X Glutamax and 1X Penicillin/streptomycin (Gibco) with the stimuli described above in the presence of Golgi Plug (BD). After incubation, cells were washed by centrifugation at 1,500 rpm for 5 min in 1X PBS, stained for 20 min on ice with antibodies CD19 (SJ25C1) and CD27, washed again, resuspended in BD Cytofix/Cytoperm for 20 minutes on ice. Cells were then washed in 1X Perm/Wash (BD) and incubated in 1X Perm/Wash with antibodies (clone): IL-6 (MQ2-13A5), TNFα (Mab11), IgG (G18-145) or isotype controls for intracellular staining for 30 min on ice. Cells were washed with 1X Perm/Wash by centrifugation at 1,500 rpm for 5 min at 4˚C, fixed by incubation in 4% PFA before data acquisition. Isotype controls were used to set gates.

### Quantification of cytokines in plasma

Plasma was thawed on ice and levels of TNFα in plasma and quantified using the U-PLEX Assay (Meso Scale Discovery, Rockville, MD). The QuickPlex SQ 120 Instrument was used to evaluate cytokine levels (Meso Scale Discovery).

### Quantification of autoreactive antibodies and total IgG in plasma

Autoreactive IgG antibodies and total IgG levels were enumerated in plasma samples as previously described (30, 31).

### Data analysis

Prism Graph-Pad Software (v 9.2.0) was used for statistical analysis. Paired t test was used to determine the significance of differences between unswitched or switched memory B cells. The significance of differences between cohorts (mild or severe infection vs. healthy controls vs immunized samples) was evaluated using one-way ANOVA or between stimulations tested *in vitro*. The significance of differences between TNFα levels with the frequency of memory populations or between the frequency of memory populations with autoreactive antibody titers was examined using a Pearson correlation. Significance was defined as p< 0.05 and the type of statistical test used is indicated in each figure legend. Each dot on the scatter plot is a single human donor. Each figure legend states the number of human donors examined in each assay.

## Results

### Reduced frequency of unswitched memory B cells with severe SARS-CoV-2 infection

**Figure 1:**
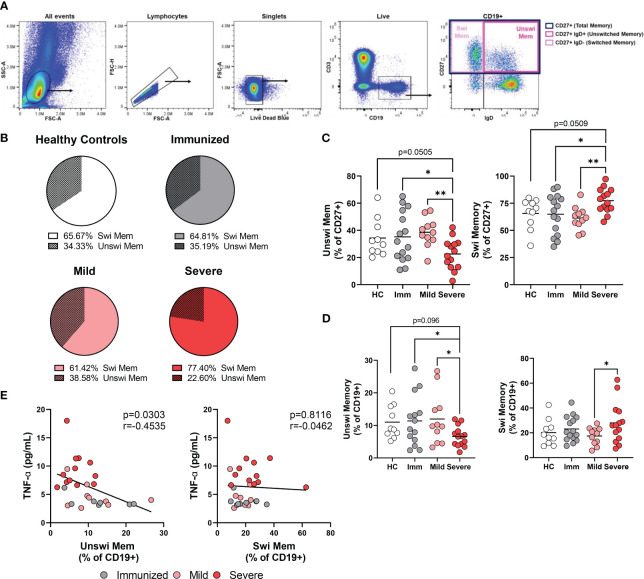
Alteration in the frequency of memory populations with Severe SARS-CoV-2 infection. **(A)** Flow plots depicting gating strategy to identify switched memory (CD19+ CD27+IgD-) and unswitched memory (CD19+ CD27+IgD+) B cells within the memory compartment (CD27+). Images from a representative mild SARS-CoV-2 subject. **(B)** Comparison of the average frequency of switched to unswitched memory B cells out of the memory compartment (CD27+) within each cohort. Sample size: Healthy controls (HC; N=10), Immunized controls (Imm; N=15), subjects with Mild (N=11) or Severe SARS-CoV-2 infection (N=14). **(C)** Frequency of switched memory or unswitched memory as a percent of the memory compartment (CD27+) or **(D)** as a percent of total B cells (CD+19). **(E)** Correlation of the frequency of unswitched or switched memory B cells as a percentage of CD19 versus levels of TNFα from plasma. Statistics: One-way ANOVA or Pearson correlation, *p<0.05, **p<0.01.

To better understand the phenotypic and functional differences between unswitched and switched memory B cells, particularly in the context of viral infection, we acquired PBMCs from healthy controls, people immunized *via* the mRNA platform against SARS-CoV-2, individuals with mild SARS-CoV-2 in the convalescent stage, and patients with severe SARS-CoV-2 who were hospitalized in the ICU at local hospitals (**Supplemental Table 1**). For this study, we evaluated total memory B cells irrespective of antigen specificity. Blood was collected between 7-507 days post booster for vaccinated individuals, between 28-305 days post positive PCR test for mildly infected individuals or between 2-35 days post admittance to hospital for severely infected subjects (**Supplemental Table 1**). The memory B cell compartment comprises unswitched memory B cells identified in human PBMCs *via* CD27 and IgD (CD27+ IgD+) expression, whereas switched memory B cells identified by expression of CD27 but not IgD (CD27+IgD-) (**Figure 1A**). In healthy controls, the unswitched memory B cell population was less frequent (~33% of CD27+ B cells) than the switched memory B cell population (~66% of CD27+ B cells; **Figure 1B**). Similarly, unswitched memory B cells were less frequent than switched memory B cells within the memory compartment of the other cohorts (**Figure 1B**).

Comparing the frequency of unswitched memory B cells in peripheral blood between cohorts, we observed a significant reduction in the proportion of unswitched memory B cells within the memory compartment during severe SARS-CoV-2 infection when compared to healthy controls, immunized or mildly infected individuals and a corresponding increase in switched memory B cells with severe infection (**Figure 1B, C**). Examination of the unswitched memory B cells within the total B cell population revealed a reduction in the percentage of unswitched memory B cells with severe SARS-CoV-2 infection when compared to mild infection, vaccination, or healthy controls (**Figure 1D**), in keeping with previous reports (22–25, 27).

Individuals with RA have been reported to harbor a reduced frequency of unswitched memory B cells, however, RA individuals who respond positively to anti-TNFα treatment display normal frequencies of unswitched memory B cells in peripheral blood (17), supporting the possibility that elevated levels of systemic TNFα may promote loss of unswitched memory B cells. We have previously reported that individuals with severe SARS-CoV-2 infection have significantly increased levels of TNFα in plasma (30). Accordingly, we compared the frequency of unswitched memory B cells with plasma levels of TNFα in the same cohorts and found that the frequency of unswitched memory B cells demonstrates a significant inverse correlation with levels of TNFα, a relationship that was not observed with switched memory B cells (**Figure 1E**).

Multiple reports provide evidence that unswitched memory B cells are protective during viral infection; the frequency of unswitched memory B cells negatively correlated with disease activity during SARS-CoV-2 infection (22), unswitched memory B cells in principal component analysis of clinical biomarkers clustered with patient discharge from the hospital (25) and, although identified by different phenotypic criteria (CD38^-^CD24^+^), unswitched memory B cells negatively correlate with symptom duration (29). Here, we compared the frequency of unswitched memory B cells from individuals with severe SARS-CoV-2 whose clinical outcome was survival versus death. These analyses did not find a significant difference in the frequency of unswitched memory between patients who survived versus those that succumbed to viral infection (**Supplemental Figure 1**), demonstrating frequency does not predict clinical outcome in our cohort.

### Expansion of the autoreactive IgD+only memory population with severe SARS-CoV-2 infection

**Figure 2:**
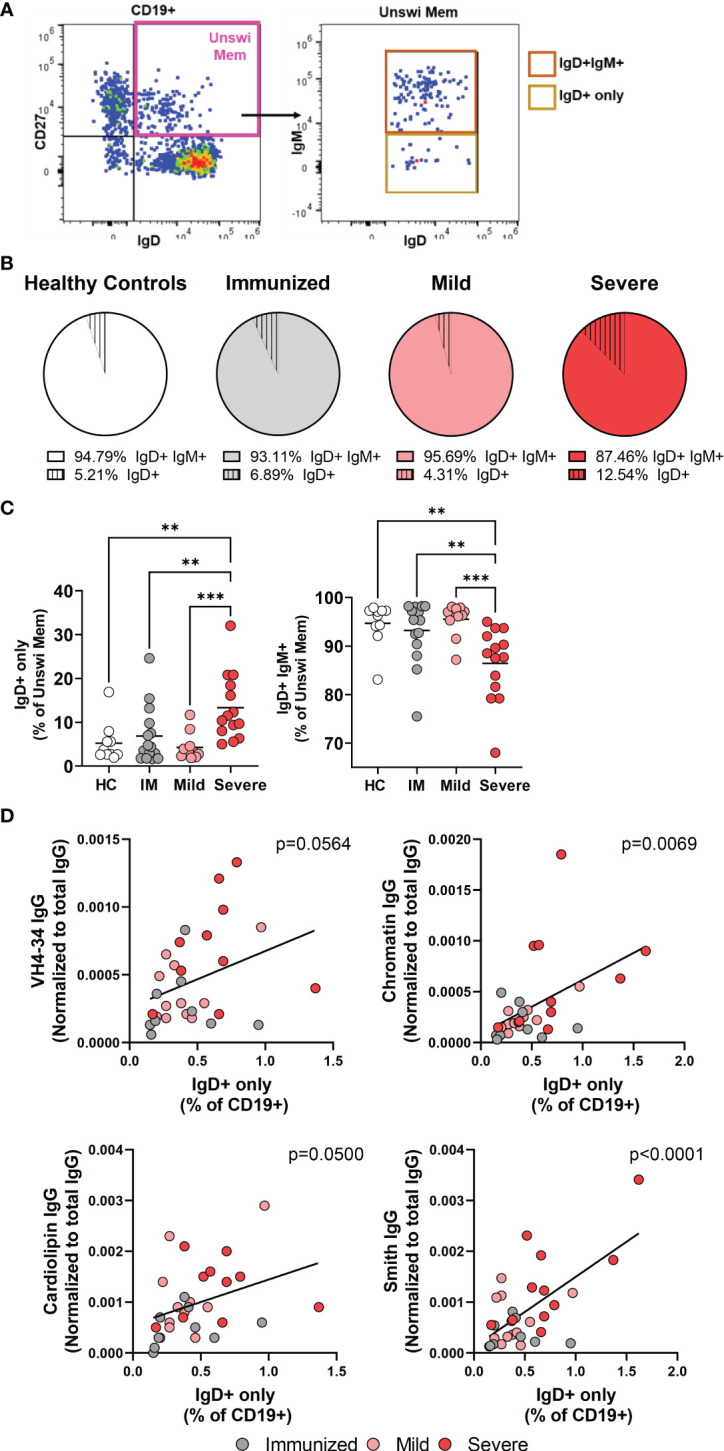
Increases in the IgD+only memory population with severe SAR-CoV-2 infection. **(A)** Representative flow plot from severe SARS-CoV-2 infection depicting the IgD+only memory population (CD19+ CD27+ IgD+ IgM-). **(B)** Comparison of the average frequency of IgD+IgM+ memory or IgD+only memory out of the unswitched memory compartment (CD27+IgD+) within each cohort. Sample size: Healthy controls (HC; N=10), Immunized controls (IM; N=15), subjects with Mild (N=11) or Severe SARS-CoV-2 infection (N=14). **(C)** Frequency of IgD+only or IgD+IgM+ memory subsets as a percent of the unswitched memory population (CD27+ IgD+). **(D)** Correlation of the frequency of IgD+only memory B cells with plasma levels of autoreactive IgG antibodies (anti-VH4-34, anti-Chromatin, anti-Smith, or anti-Cardiolipin) normalized to total IgG. Statistics: One-way ANOVA or Pearson correlation, **p<0.01, ***p<0.001.

The unswitched memory B cell population (CD27+ IgD+) includes two subpopulations; the IgD+IgM+ memory subset and the IgD+IgM- subset which has lost expression of IgM and is termed ‘IgD+only’ memory cells (**Figure 2A**). It has been reported that IgD+only memory cells have somatically mutated V genes indicative of antigen experience (32), however, their role in viral infection has not been evaluated to date. In healthy controls, we found that amongst the unswitched memory B cell population, the IgD+only memory subset is approximately 5% and much less frequent than the IgD+IgM+ subset (**Figure 2B**). Similarly, immunized individuals and mild or severely infected subjects also harbor an IgD+only memory subset that is less frequent than the IgD+IgM+ subset within the unswitched memory population (**Figure 2B**). Notably, however, we observed a significant increase in the IgD+only memory B cell population with severe SARS-CoV-2 infection representing approximately 12.5% and a corresponding decrease in the IgD+IgM+ subset out of the unswitched memory population (**Figure 2C**). These results indicate that viral infection promotes the expansion of the IgD+only memory subset or, conversely, a loss of IgD+IgM+ memory B cells.

We and others have reported increased titers of autoreactive antibodies with severe SARS-CoV-2 infection demonstrating viral infection promotes a breach in B cell immunological tolerance (30, 33–36). IgD+only memory cells from healthy human controls are enriched in high-affinity autoreactive BCRs with specificity towards anti-nuclear antigens, ss/dsDNA, LPS, and insulin (7). Given that we observe an increased frequency of IgD+only cells, we asked whether there was an association between this subset and SARS-CoV-2 infection-induced autoantibody production. Importantly, we find a significant correlation between the frequency of IgD+only cells within the total B cell population and plasma titers of not only VH4-34 IgG but also with levels of IgG anti-chromatin, anti-Smith, and anti-cardiolipin (**Figure 2D**). Furthermore, neither the frequency of CD27+IgD+ unswitched memory B cells nor CD27+IgD- switched memory B cells correlated with levels of autoantibodies (**Supplemental Figure 2**), highlighting the specificity of the relationship between the presence of (CD27+IgD+IgM-) IgD+only memory cells and autoantibody titers. These data demonstrate that as the frequency of IgD+only cells increases with SARS-CoV-2 infection, autoreactive antibody titers also increase, such that IgD+-only B cells appear to be a novel contributor to autoreactive antibody production during viral infection.

Since severe SARS-CoV-2 infection resulted in an alteration of the unswitched memory compartment with an increase in the percentage of IgD+only B cell population and a concomitant decrease in IgM+IgD+ B cells (**Figure 2**), we questioned whether the switched memory population subsets were also altered with severe viral infection, thus we examined the total switched memory population, rather than antigen-specific B cells. The switched memory population is comprised of memory B cells expressing immunoglobulin isotype class IgM (pre-switched) or IgG (**Supplemental Figure 3A**) and a substantial subset of IgM-IgG- which are IgA-expressing memory B cells (**Supplemental Figure 3B**). Healthy controls harbor IgG-expressing and IgM-IgG- (IgA+) memory B cells that are more frequent relative to IgM-expressing memory B cells (**Supplemental Figure 3C**). A similar composition was observed in switched memory subsets from vaccination or mild infection (**Supplemental Figure 3C**). However, comparing switched memory subsets between the cohorts revealed that there is a significant reduction in IgG+ memory B cells during severe SARS-CoV-2 infection compared to healthy controls and mildly infected individuals, and a significant increase in IgM-IgG- (IgA+) memory B cells when comparing mild to severe SARS-CoV-2 infection (**Supplemental Figure 3D**). Together these data demonstrate that severe SARS-CoV-2 infection modulates the memory compartment immunoglobulin isotype.

### Activation and loss of inhibition of unswitched memory B cells with severe SARS-CoV-2 infection

**Figure 3:**
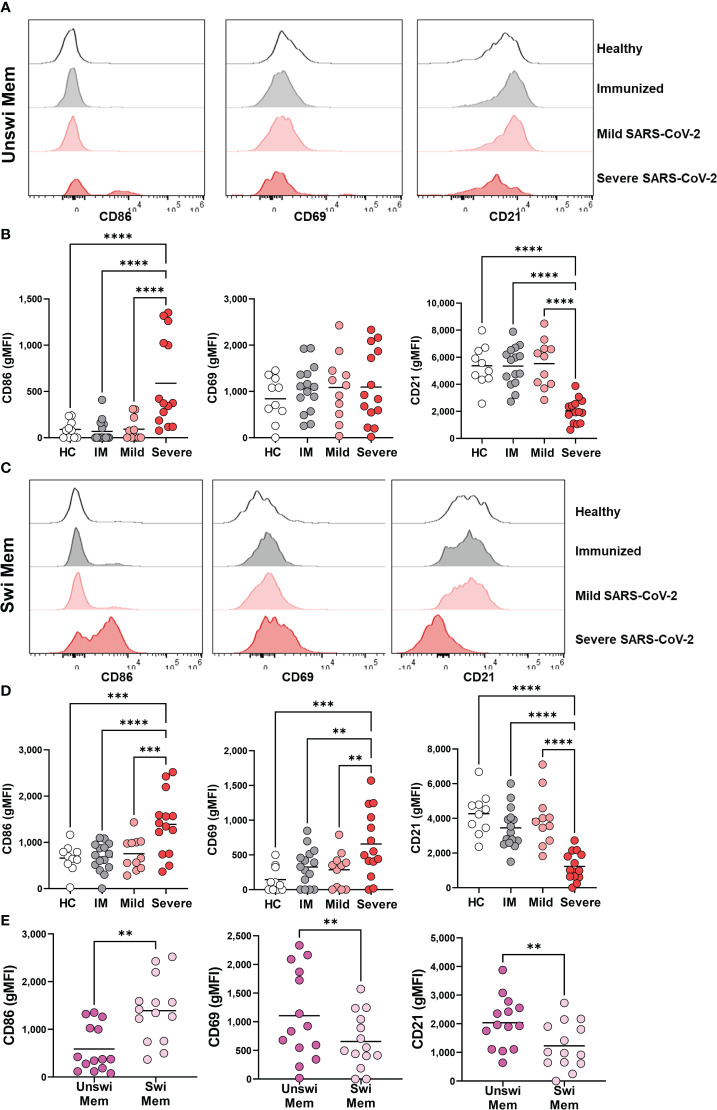
Activation of Unswitched and Switched Memory B cells with Severe SARS-CoV-2 infection. **(A)** Representative histograms displaying an expression of CD86, CD69, and CD21 on unswitched memory B cells or **(C)** switched memory B cells from healthy control, immunized control, and mild or severe SARS-CoV-2 infected subjects. **(B)** Quantification of expression level (geometric MFI) of CD86, CD69, and CD21 on unswitched memory B cells or **(D)** switched memory B cells from each cohort. Sample size: Healthy controls (HC; N=10), Immunized controls (IM; N=15), subjects with Mild (N=11) or Severe SARS-CoV-2 infection (N=14). **(E)** Comparison of unswitched to switched memory B cells for expression of CD86, CD69, and CD21 from severely infected individuals. Statistics: One-way ANOVA or paired t test, **p<0.01, ***p<0.01, ****p<0.0001.

To evaluate how viral infection modulates the activation status of unswitched memory B cells, we evaluated the expression of the CD86 and CD69 activation antigens as well as the CD21 co-receptor on total memory B cells (rather than antigen-specific B cells). These analyses demonstrated a significant increase in expression of CD86, but not CD69, and a significant reduction of CD21 on unswitched memory B cells with severe SARS-CoV-2 infection (**Figures 3A, B**), phenotypic changes indicative of an activated population. Similarly, we observed that switched memory B cells had significantly increased expression of CD86 and a significant loss of CD21 with severe SARS-CoV-2 infection compared to mild infection, vaccination, or healthy controls (**Figures 3C, D**). Switched memory B cells also displayed a significant increase in the expression of CD69 with severe SARS-CoV-2 infection (**Figures 3C, D**). Switched memory B cells were observed to have higher expression of CD86 compared to unswitched memory B cells in severe infection, whereas unswitched memory B cells displayed higher expression levels of CD21 than switched memory (**Figure 3E**). Interestingly, despite the increase in CD69 expression on switched memory B cells with SARS-CoV-2 infection, expression of this protein never reached the levels of CD69 expression on unswitched memory (**Figure 3E**), such that unswitched memory B cells reside at a higher activation state in general. These data indicate that both the unswitched and switched memory B cell compartments are highly activated with severe viral infection.

**Figure 4:**
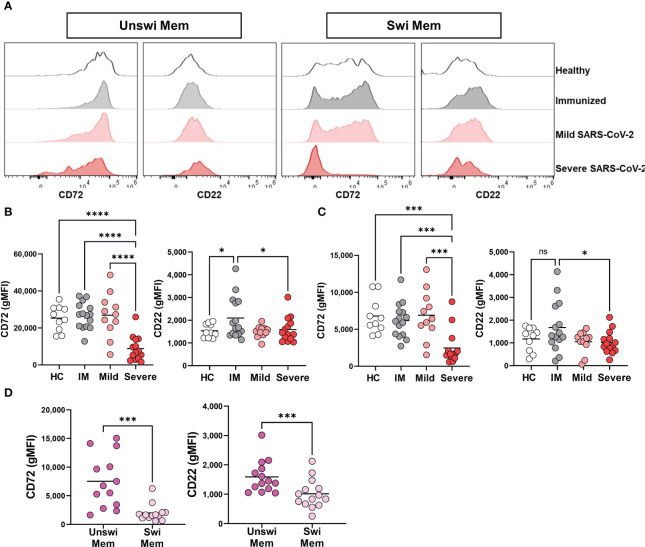
Loss of inhibition on Unswitched and Switched Memory B cells with Severe SARS-CoV-2 infection. **(A)** Representative histograms displaying expression of CD72 and CD22 on unswitched or switched memory B cells from a healthy control (HC), immunized control (IM), mild or severe SARS-CoV-2 infected subject. **(B)** Quantification of expression level (geometric MFI) of CD72 and CD22 on unswitched or **(C)** switched memory B cells from each cohort. Sample size: Healthy controls (HC; N=10), Immunized controls (IM; N=15), subjects with Mild (N=11) or Severe SARS-CoV-2 infection (N=14). **(D)** Comparison of unswitched to switched memory B cells from severely infected SARS-CoV-2 individuals. Statistics: One-way ANOVA or paired t test, ns, not significant *p<0.05, ***p<0.01, ****p<0.0001.

Because severe viral infection results in phenotypically activated unswitched and switched memory B cells (**Figure 3**), we sought to determine if activation of the memory B cell populations also coincides with a reduction in inhibitory receptors by memory B cells through evaluation of CD72 and CD22. Unswitched memory B cells had a significant reduction in CD72 expression during severe SARS-CoV-2 infection when compared to mild infection, vaccination, or healthy controls (**Figures 4A, B**). A similar reduction in the expression of CD72 was observed on switched memory B cells from severely infected SARS-CoV-2 patients (**Figures 4A, C**). Interestingly, immunization to SARS-CoV-2 with mRNA vaccination increased the expression level of CD22 on both unswitched and switched memory B cells as compared to healthy controls such that there was a significant difference in CD22 expression between immunized and severely infected individuals (**Figures 4A, C**). Comparison of unswitched to switched memory B cells revealed that unswitched memory B cells have higher expression of CD72 and CD22 than switched memory B cells during severe SARS-CoV-2 infection (**Figure 4D**), indicating differential regulation of these populations.

### Enhanced BCR signaling of unswitched memory B cells with severe SARS-CoV-2 infection

**Figure 5:**
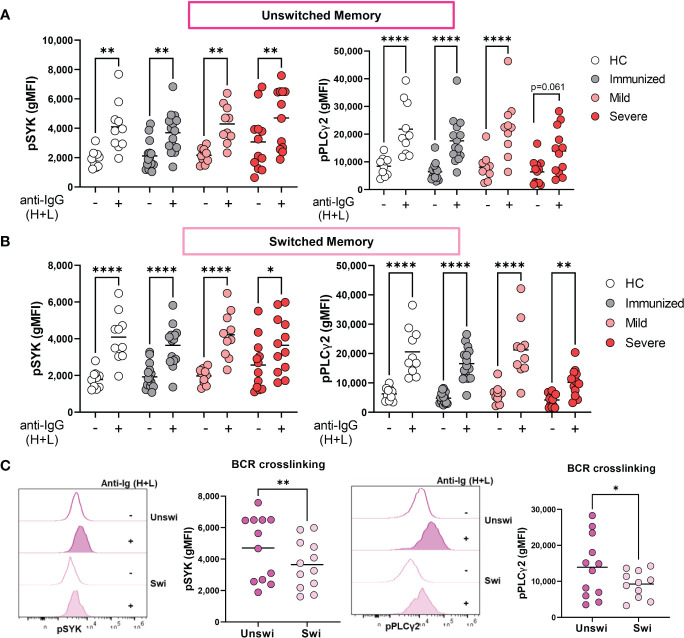
Enhanced BCR signaling of Unswitched Memory B cells during Severe SARS-CoV-2 infection. **(A)** Quantification of expression levels of pSYK and pPLCγ2 (gMFI: geometric mean fluorescent intensity) on unswitched or **(B)** switched memory B cells from healthy controls (HC, N=10), individuals immunized against SARS-CoV-2 (N=15), or individuals with mild (N=10) or severe SARS-CoV-2 infection (N=12) without **(-)** or with (+) stimulation by 10μg/mL anti-IgG (H+L) F(ab’)2 for 5 min. **(C)** Representative histograms and quantification comparing levels of pSYK and pPLCγ2 in unswitched and switched memory B cells from severe SARS-CoV-2 infection after stimulation with 10μg/mL anti-IgG (H+L) F(ab’)2 for 5 min. Statistics: One-way ANOVA or paired t test, *p<0.05, **p<0.01, ****p<0.0001.

We have previously reported that Double Negative B cells signal *via* the BCR during viral infection (31); however, the BCR signaling capacity of unswitched memory B cells during viral infection has not been reported. To address this, we stimulated PBMCs with and without anti-human Ig to induce BCR signaling and assessed expression levels of phospho-SYK (pSYK) and pPLCγ2, effector molecules in the BCR signal transduction cascade. The results from these analyses revealed that BCR signal transduction is intact in unswitched memory B cells from all cohorts as evidenced by significant increases in pSYK and pPLCγ2 levels upon BCR stimulation (**Figure 5A**). Similar results were also observed for switched memory B cells (**Figure 5B**). Thus, despite the activated and dysregulated state of memory B cells with severe SARS-CoV-2 infection, they maintain the ability to signal through the BCR. Importantly, upon comparison of unswitched to switched memory B cells from severe SARS-CoV-2 infection, unswitched memory B cells express higher levels of pSYK and pPLCγ2 after BCR stimulation than switched memory B cells (**Figure 5C**), (which was surprising given higher expression of inhibitory receptors CD72 and CD22 on unswitched memory B cells) demonstrating that unswitched memory B cells are better poised to respond to antigen recognition than switched memory B cells during severe viral infection.

### B cells from mild SARS-CoV-2 infection produce proinflammatory cytokines

**Figure 6:**
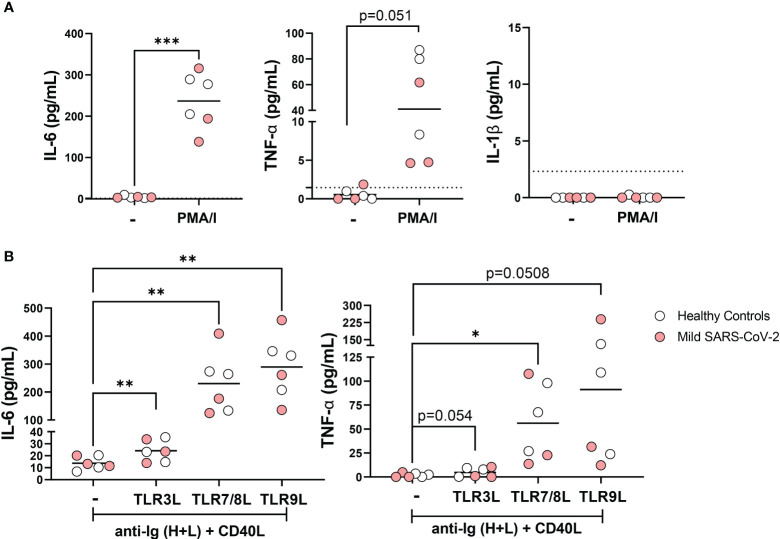
Secretion of pro-inflammatory cytokines IL-6 and TNFα by B cells in response to viral TLR ligands. **(A)** B cells were purified from healthy controls (white, N=3) or subjects with mild SARS-CoV-2 infection (red, N=3) then stimulated *in vitro* overnight with 50ng/mL PMA+ 1ug/mL Ionomycin or unstimulated control (-) and levels of IL-6, IL-1B, and TNFα were measured in cell culture supernatant. **(B)** B cells were purified from healthy controls (white, N=3) or subjects with mild SARS-CoV-2 infection (red, N=3) then stimulated *in vitro* overnight with 10ug/mL anti-Ig (H+L) F(ab)’2 + 100ng/mL CD40L with either 1ug/mL TL3RL (Poly I:C), 1ug/mL TLR7/8L (R848), or 2.5ug/mL TLR9L (CpG) and secretion of IL-6 and TNFα were measured in cell culture supernatant. Statistics: paired t test or one way ANOVA, *p<0.05, **p<0.01, ***p<0.001.

B cells from healthy individuals are capable of producing cytokines in response to TLR ligand stimulation (21, 37) and we and others have previously reported that individuals with severe SARS-CoV-2 infection have increased levels of pro-inflammatory cytokines (30, 38, 39). Accordingly, we assessed whether B cells could contribute to the inflammatory milieu during viral infection. To address this, we purified total B cells from a small subset of healthy controls and mild SARS-CoV-2 infection, stimulated the cells *in vitro*, and measured cytokine production in the supernatant. Stimulation of total B cells with PMA and ionomycin for 18 hours significantly increased the secretion of cytokines IL-6 and TNFα, but not IL-1β (**Figure 6A**). To determine if B cells participating in immune response (after BCR stimulation and T cell help) could secrete these cytokines in response to viral-specific TLR ligands, we stimulated purified B cells from healthy controls and mild SARS-CoV-2 infection with ligands specific for TLR3, TLR7/8, or TLR9 in the presence of polyclonal BCR crosslinking and T cell help. These results demonstrated that B cells can contribute to the inflammatory cytokine milieu by secreting pro-inflammatory IL-6 and TNFα, in response to all three viral TLR ligands (**Figure 6B**). We did not see differences in the amount of IL-6 or TNFα when comparing healthy controls with mild SARS-CoV-2 infection (**Supplemental Figure 4**). Interestingly, the highest levels of secreted cytokines were detected in stimulations with TLR7/8L or TLR9L as compared to TLR3L (**Figure 6B**) with the highest levels of IL-6 or TNFα secreted in response to TLR9L, even above levels from PMA and ionomycin stimulation (**Figure 6**). Together these data indicate that B cells are able to and likely contribute to the pro-inflammatory cytokine milieu during viral infection.

### IL-6 and TNFα production by Unswitched and Switched Memory B cells in response to viral TLR ligand stimulation

**Figure 7:**
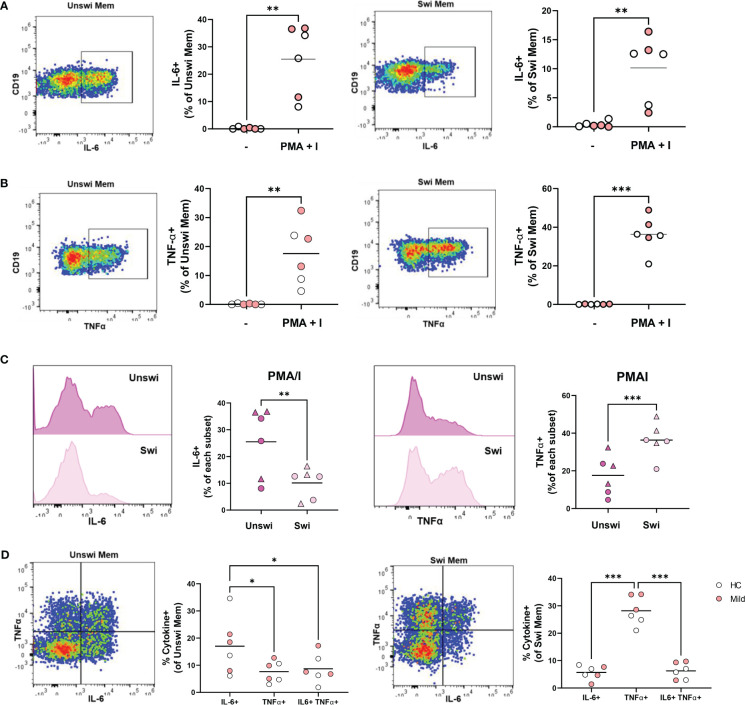
IL-6 and TNFα production by Unswitched and Switched Memory B cells in response to PMA/I stimulation. **(A)** Representative flow plots from a mild SARS-CoV-2 subject and quantification of IL-6+ or **(B)** TNFα+ unswitched or switched memory B cells from 4-hour *in vitro* stimulation of PBMCs from healthy controls (white, N=3) or subjects with mild SARS-CoV-2 infection (red, N=3) with 50ng/mL PMA+ 1ug/mL Ionomycin or unstimulated control (-). **(C)** Histograms from a mild SARS-CoV-2 subject and quantification of the comparison of the frequency of IL-6+ or TNFα+ unswitched to switched memory B cells upon PMA/I stimulation from healthy controls (circle, N=3) or subjects with mild SARS-CoV-2 infection (triangle, N=3). **(D)** Representative flow plots from a mild SARS-CoV-2 subject and quantification of the frequency of mono-or dual cytokine positive unswitched or switched memory B cells upon PMA/I stimulation. Statistics: paired t test or one-way ANOVA, *p<0.05, **p<0.01, ***p<0.001.

Although it has been reported that total (unswitched and switched) memory B cells (CD27+) from healthy controls produce more cytokines than naive B cells (21), we asked whether unswitched memory B cells *per se* were capable of producing cytokines. Stimulation of PBMCs with PMA and ionomycin from a subset of healthy controls and mild SARS-CoV-2 infection resulted in a significantly increased proportion of IL-6-expressing unswitched memory B cells (**Figure 7A**), that were predominantly IgD+IgM+ unswitched memory B cells rather than the IgD+only subset (**Supplemental Figure 5A**). Similarly, PMA and ionomycin stimulation significantly increased the frequency of IL-6-expressing switched memory B cells (**Figure 7A**) and the majority of the IL6-expressing switched memory cells were IgG+ followed by a fraction of IgM-IgG- (IgA+) cells in both healthy controls and mildly-infected individuals (**Supplemental Figure 5B**). Examination of TNFα expression revealed that both unswitched and switched memory B cells produce TNFα in response to PMA and ionomycin stimulation (**Figure 7B**), with the dominant fraction of TNFα-expressing unswitched memory IgD+IgM+ and switched memory IgM-IgG- (IgA+) in both healthy controls and mildly-infected individuals (**Supplemental Figures 5A, B**).

Comparing unswitched to switched memory B cells, the frequency of IL-6-expressing memory B cells were significantly higher for unswitched memory B cells compared to switched memory B cells after stimulation with PMA and ionomycin stimulation (**Figure 7C**), whereas there was a significantly higher frequency of TNFα- expressing switched memory compared to unswitched memory (**Figure 7C**). These findings demonstrate unswitched memory cells are more poised to produce the proinflammatory IL-6 cytokine, whereas, switched memory B cells are better poised to produce TNFα. We further queried whether memory B cells were polyfunctional in their ability to produce both IL-6 and TNFα. Although polyfunctional IL-6- and TNFα-expressing unswitched memory and switched memory B cells were observed, the majority of each population from healthy controls and mild SARS-CoV-2 infection produced a single cytokine rather than both with unswitched memory B cells mostly producing IL-6 and switched memory B cells mostly expressing TNFα (**Figure 7D**). Together these results indicate that unswitched memory B cells are capable of producing pro-inflammatory cytokines.

**Figure 8:**
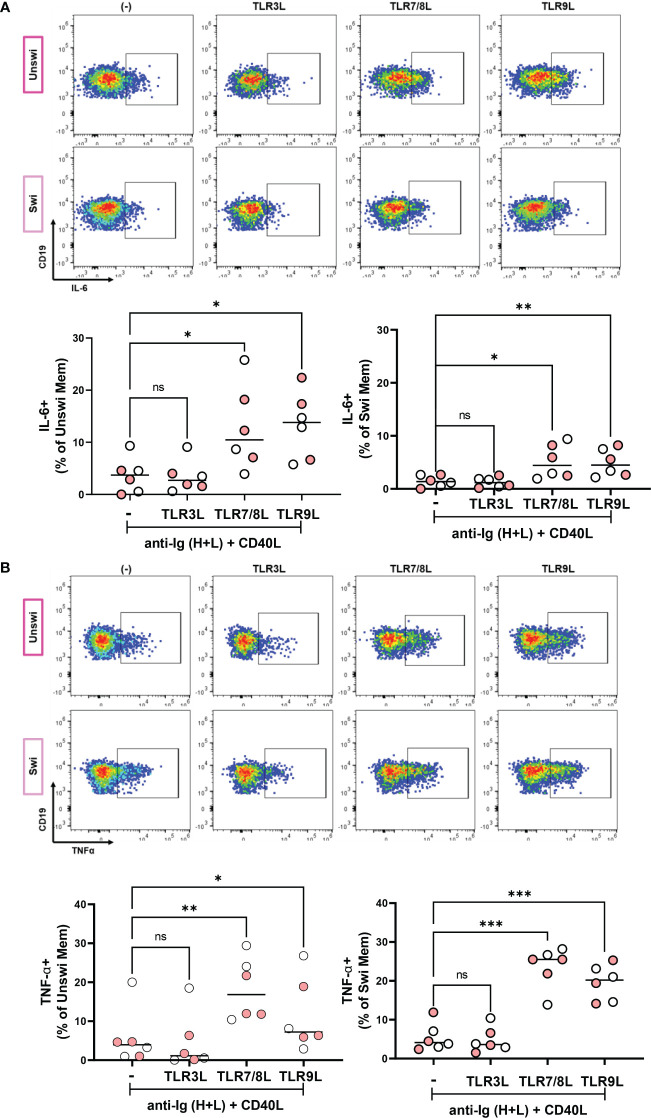
IL-6 and TNFα production by Unswitched and Switched Memory B cells in response to viral TLR ligand stimulation. **(A)** Representative flow plots from a mild SARS-CoV-2 subject and quantification of IL-6+ or **(B)** TNFα+ unswitched or switched memory B cells from healthy controls (white, N=3) or subjects with mild SARS-CoV-2 infection (red, N=3) upon PBMC 4 hour *in vitro* stimulation with 10ug/mL anti-Ig (H+L) F(ab)’2 + 100ng/mL CD40L and with either 1ug/mL TL3RL (Poly I:C), 1ug/mL TLR7/8L (R848), or 2.5ug/mL TLR9L (CpG). Statistics: one way ANOVA, ns not significant, *p<0.05, **p<0.01, ***p<0.001.

We next determined if unswitched and switched memory B cells participating in an immune response upon BCR engagement and provision of T cell help could produce IL-6 and TNFα in response to viral TLR ligands. Thus, we stimulated PBMCs from healthy controls and mild SARS-CoV-2 infection with ligands specific for TLR3, TLR7/8, or TLR9 in the presence of polyclonal BCR crosslinking and T cell help. These data revealed that stimulation with TLR7/8L and TLR9L, but not TLR3L, significantly increased the frequency of IL-6-expressing unswitched and switched memory B cells (**Figure 8A**). Finally, stimulation with TLR7/8L or TLR9L but again not TLR3L significantly increased the frequency of TNFα-expressing unswitched or switched memory B cells (**Figure 8B**). We did not see differences in the frequency of IL-6 or TNFα- expressing unswitched or switched memory B cells when comparing healthy controls to individuals with mild SARS-CoV-2 infection (**Supplemental Figures 5C, D**). Together these data indicate that memory B cells differentially respond to viral TLR ligands (more poised to respond to ssRNA or unmethylated DNA rather than dsRNA) and demonstrate that unswitched memory B cells likely contribute to the pro-inflammatory cytokine milieu during viral infection.

## Discussion

This study examined the frequency, phenotype, and functionality of unswitched and switched memory B cells in populations with severe or mild COVID-19 infection, those who had been vaccinated, or healthy controls. Our results corroborate previously reported findings, indicating a significant reduction in the frequency of unswitched memory B cells with severe SARS-CoV-2 infection. We showed that this reduction in unswitched memory B cells is significantly associated with elevated levels of TNFα during viral infection. Importantly, we observed an expansion of the IgD+only subset of unswitched memory B cells that correlated with elevated levels of autoantibodies with severe SARS-CoV-2 infection. We show that both unswitched and switched memory B cells are highly activated, and have lost expression of inhibitory receptors, and the unswitched memory population has enhanced BCR signaling with viral infection. Furthermore, we demonstrate that memory B cells secrete cytokines in response to viral ligands and specifically unswitched memory B cells are dominant producers of IL-6 whereas switched memory B cells primarily produce TNFα. These data reflect global changes in the total memory B cell populations rather than antigen-specific memory B cells. Together, these data provide evidence that the unswitched memory population is highly altered with severe SARS-CoV-2 infection and highlight the novel role that unswitched memory B cells may contribute to the inflammatory milieu during viral infection.

A reduction in the unswitched memory B cell population has been reported in multiple autoimmune diseases including SLE (15, 16), RA (17) and Sjögren’s syndrome (14). Similarly, multiple studies on severe SARS-CoV-2 infection have also reported a reduction in unswitched memory B cells (22–27). Here in our study, we confirm the reported reduction of unswitched memory B cells out of the total B cell population in this study, and further report a fluctuation in the memory compartment whereby there is a reduction in the frequency of unswitched memory B cells and a corresponding increase in the switched memory population with severe SARS-CoV-2 infection. Whether these changes during severe viral infection reflect bona fide class-switch recombination by unswitched memory B cells to become switched memory B cells remains to be studied. One limitation of this study is that we focused on circulating unswitched memory B cells, while little is still known about tissue resident unswitched memory B cells after SARS-CoV-2 infection. It is also possible that the observed changes in the frequency of circulating B cell memory population reflect emigration from the peripheral blood into tissue and lymph nodes rather than a true loss of B cells within a subset.

Two groups report that in the convalescent stage after mild infection with SARS-CoV-2, no differences in the frequency of unswitched memory B cells were observed compared to healthy controls (28, 29). In keeping with these reports, we did not find any difference in the frequency of unswitched memory B cells with mild infection and indicating this population has minimal fluctuation with mild viral infection in contrast to the striking changes observed with severe viral infection where the levels of systemic proinflammatory cytokines are much higher. It is also unclear how infection with other viruses modulates the unswitched memory population although one group reported that African women with HIV infection have an increase in the frequency of unswitched memory B cells, but this population returns to levels found in healthy controls upon antiretroviral treatment (40), suggesting that control of the virus allows for the reestablishment of normal levels of unswitched memory B cells. Together these studies indicate that viral infection modulate the unswitched memory population.

The contribution to or protection from SARS-CoV-2 infection by unswitched memory B cells remains to be determined. Although identified by differing surface markers, one study reported that a higher frequency of IgM-memory (including IgM+ only and unswitched memory) B cells in convalescent donors correlated with reduced symptom duration upon mild SARS-CoV-2 infection (29) indicating a protective response by this subset. Another group reported that a lower frequency of unswitched memory B cells negatively correlated with systemic levels of CRP and disease severity (22). Furthermore, the lower frequency of unswitched memory B cells clustered with the clinical outcome of discharge from the hospital (25). In contrast, one group reported that a lower frequency of switched memory but not unswitched memory B cells was a risk factor for severity score and mortality with severe infection (41), implying that switched memory B cells prevent the clinical outcome of worse disease and death with SARS-CoV-2 infection. Here in our study, we compared the frequency of unswitched memory cells to the outcome of severe viral infection (survival or death) but did not see any significant correlations, thus we are unable to provide supporting evidence that having more unswitched memory B cells enhances protection against viral infection in our cohort.

We and others have reported increased autoreactive antibody titers during severe SARS-CoV-2 infection (30, 33–35). In the context of the SLE autoimmune disease, a negative correlation between levels of autoantibodies and the presence of unswitched memory B cells has been reported whereby SLE patients with more autoreactive antibodies have fewer unswitched memory B cells (15, 16). Further, a negative correlation between levels of anti-SSa autoantibodies and frequency of unswitched memory B cells was also reported in Sjögren’s syndrome (14), indicating this population may be protective against autoimmunity. However, 9G4-expressing B cells (identifying the autoreactive VH4-34 idiotype) are more frequently found amongst unswitched memory B cells from SLE patients than healthy controls (16), although this may be indicative of more widespread defects in immunological tolerance with SLE since increased frequencies of 9G4-expressing cells have been reported for other B cell subsets as well (16, 42). In the current study, we did not observe any significant correlations between the frequency of all unswitched memory B cells and levels of autoreactive antibodies in the plasma of study participants. In accordance with these findings, unswitched memory B cells have been shown to display reduced levels of autoreactive BCRs compared to naive B cells from healthy controls (43). Another study demonstrated that unswitched memory B cells from RA have a reduced capacity to produce autoreactive IgM (17). We note, however, that the IgD+-only subset of unswitched memory B cells are enriched in autoreactive and polyreactive BCRs (7), and in our study we found an expansion of the IgD+only subset within the unswitched memory population during severe SARS-CoV-2 infection and a positive correlation of the frequency of IgD+only cells with levels of autoreactive antibodies, suggesting this minor subset may contribute to the production of autoantibodies during viral infection whereas the rest of the memory compartment does not.

Unswitched memory B cells were reported to be more activated in SLE patients as shown by increased expression of CD86 (16). The results from our experiments agree as we found that unswitched memory B cells expressed higher levels of CD86 during severe SARS-CoV-2 infection. Loss of CD21 expression is associated with B cell activation (44) and unswitched memory B cells in healthy human peripheral blood and spleen are CD21^hi^ (9). Here, we see that CD21 expression is reduced on unswitched memory B cells with severe infection, further supporting the activated phenotype. In regards to BCR signal transduction, it was reported that a quick burst of SYK phosphorylation in IgM+-expressing B cells (which includes unswitched memory B cells) occurred upon BCR crosslinking as compared to IgG-expressing B cells such that IgM-expressing B cells have faster kinetics downstream of the BCR and a lower threshold for response upon BCR cross linking (45). Additionally, human marginal zone like (including unswitched memory and IgM+ only switched memory) B cells have enhanced BCR signaling responses above naive B cells (increased percent of pERK1/2 upon BCR crosslinking) (46), suggesting marginal zone like B cells are more responsive to B cell activation. These reports are in line with our results revealing higher expression of pSYK and pPLCγ2 in unswitched memory B cells compared to switched memory B cells that indicate enhanced BCR activation by the unswitched memory population. It was reported that the pSYK induced in IgG-expressing B cells lasted for a longer period, which is indicative of a more durable response to BCR activation, possibly due to reduced expression of negative regulators such as the phosphatase CD22 phosphates (45). In keeping with this possibility, we report higher expression of CD22 in unswitched memory B cells compared to switched memory B cells suggesting that although unswitched memory B cells have enhanced response to BCR crosslinking, this population in turn has enhanced negative regulation. These results demonstrate that severe viral infection alters the phenotype and function of unswitched memory B cells.

IL6 gene expression was previously reported to be 2-fold higher in unswitched memory B cells from the blood and spleen compared to switched memory B cells based on RNAseq analysis, implying unswitched memory B cells are more poised to produce this pro-inflammatory cytokine (9). Our findings provide further evidence for this conclusion as we observed a higher frequency of IL-6-expressing unswitched memory B cells compared to switched memory B cells in response to TLR ligand stimulation. Upon disease remission post-treatment in both SLE (15) and in RA (17), the frequency of unswitched memory B cells reverted to normal levels, and importantly patients responding to anti-TNFα treatment had a rebound in this cell population (17), suggesting the TNFα pro-inflammatory cytokine drives the loss of unswitched memory B cells in peripheral blood. Interestingly, we observed a negative correlation between levels of TNFα in the plasma and frequency of unswitched memory cells providing evidence that TNFα may promote the loss of unswitched memory B cells with viral infection. Given the disruption to lymph node and splenic germinal centers associated with excessive levels of TNFα during COVID-19 (47), it is tempting to speculate that TNFα-induced defects of the lymph node architecture with viral infection lead to a reduction in the development of circulating unswitched memory B cells. Furthermore, since we demonstrate here that total B cells secrete pro-inflammation cytokines in response to viral ligands, B cells themselves may likely contribute to disease pathology during SARS-CoV-2 infection.

In summary, we provide evidence that the unswitched memory B cell population is changed during severe SARS-CoV-2 infection. Importantly, these findings also imply that the IgD+only subset may contribute to autoreactive antibody production and that unswitched memory B cells may promote inflammation during severe viral infection through the production of pro-inflammatory cytokines.

## Data availability statement

The raw data supporting the conclusions of this article will be made available by the authors, without undue reservation.

## Ethics statement

The studies involving human participants were reviewed and approved by The Colorado Multiple Institutional Review Board (COMIRB) at the University of Colorado School of Medicine and National Jewish Health approved the use of human PBMCs and plasma. This study was performed under the Declaration of Helsinki. The patients/participants provided their written informed consent to participate in this study.

## Author contributions

MC and RT designed the study, interpreted the data, and wrote the manuscript. MC recruited vaccinated and mild SARS-CoV-2 infected subjects, performed experiments, and analyzed the data. AS assisted in performing experiments. RP provided expertise and samples. KL, JM, KM, WJ, and JB provided severe SARS-CoV-2 samples. All authors reviewed the manuscript. All authors contributed to the article and approved the submitted version.
