# Supplementary material for: Activation and pro-inflammatory cytokine production by unswitched memory B cells during SARS-CoV-2 infection
Source: Front Immunol. 2023 Aug 10;14:1213344. doi: 10.3389/fimmu.2023.1213344 (PMC10449608; doi:10.3389/fimmu.2023.1213344)
Supplement: Supplementary file 1 [file Presentation_1.pdf]

Supplemental Material for

Activation and Pro-inflammatory Cytokine Production by Unswitched Memory B cells during SARS-CoV-2 infection

Moriah J. Castleman,[1*] Adriana Luna Santos, [1] Kelsey E. Lesteberg, [1,2] James P. Maloney,[3] William J. Janssen,[4,5] Kara J. Mould,[4,5] J. David Beckham, [1,2,6] Roberta Pelanda, [1] and Raul M. Torres[1*]

[1]Department of Immunology and Microbiology, University of Colorado School of Medicine, Aurora, Colorado, USA.

[2]Department of Medicine, Division of Infectious Disease, University of Colorado School of Medicine, Aurora, Colorado, USA.

[3]Department of Medicine, Division of Pulmonary Sciences and Critical Care Medicine, University of Colorado School of Medicine, Aurora, Colorado, USA.

[4] Department of Medicine, National Jewish Health, Denver, Colorado, USA.

[5] Department of Medicine, University of Colorado, Aurora, Colorado, USA.

[6] Rocky Mountain Regional VA, Medical Center, Aurora, Colorado, USA.

*Corresponding authors: Moriah J. Castleman, Moriah.Castleman@cuanschutz.edu and Raul Torres, Raul.Torres@cuanschutz.edu

Cohort characteristics					
**Parameter**	**Measurement**	**Healthy**	**Immunized**	**Convalescent (Mild)**	**Severe**
Age (years)	Average (range)	42.6 (24-70)	54.1 (23-82)	40.2 (23-75)	64 (40-91)
Sex	% F (Sample size)	50 (10)	53.3 (15)	50 (12)	53.3 (15)
**Time (days) of blood collection post event**					
Primary vaccination	Average (range)		128 (10-535)		
Booster	Average (range)		114 (7-507)		
2nd booster	Average (range)		155 (126-193)		
Positive PCR test	Average (range)			83 (28-305)	
Admittance to hospital	Average (range)				11.2 (2-35)

Supplemental Table 1. Demographics of individuals examined in this study. Four cohorts of individuals were examined in this study including; healthy controls, those immunized against SARS-CoV-2 with mRNA platform vaccine, those with mild SARS-CoV-2 infection not requiring hospitalization in the convalescent stage, and those with severe SARS-CoV-2 infection requiring hospitalization.

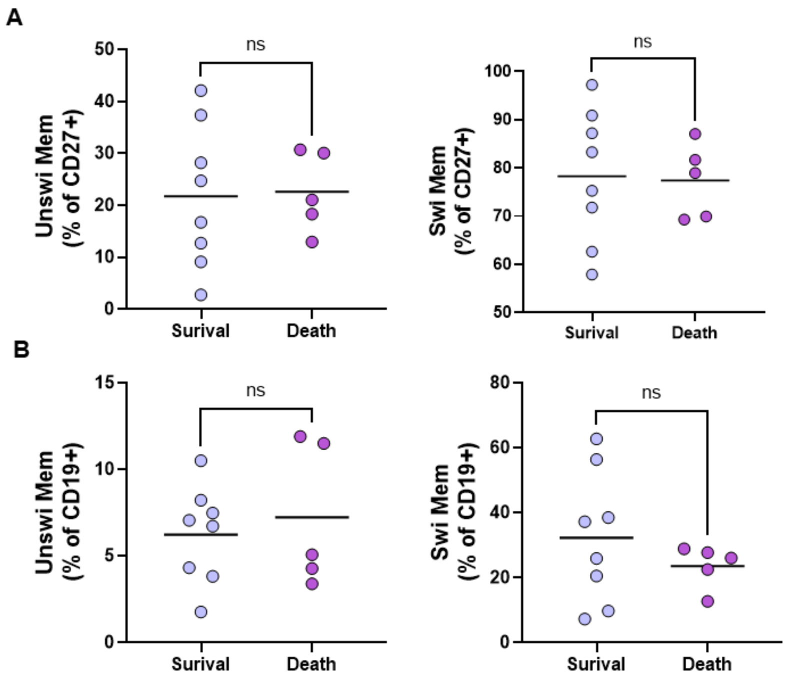

Supplemental Figure 1. Frequency of memory populations do not predict clinical outcome. (A) Frequency of unswitched or switched memory B cells as a percentage of memory (CD27+) or (B) as a percentage of total B cells (CD19+) graphed based on survival or death during hospitalization with severe SARS-CoV-2 infection (N=13). Statistics: unpaired t test, ns not significant

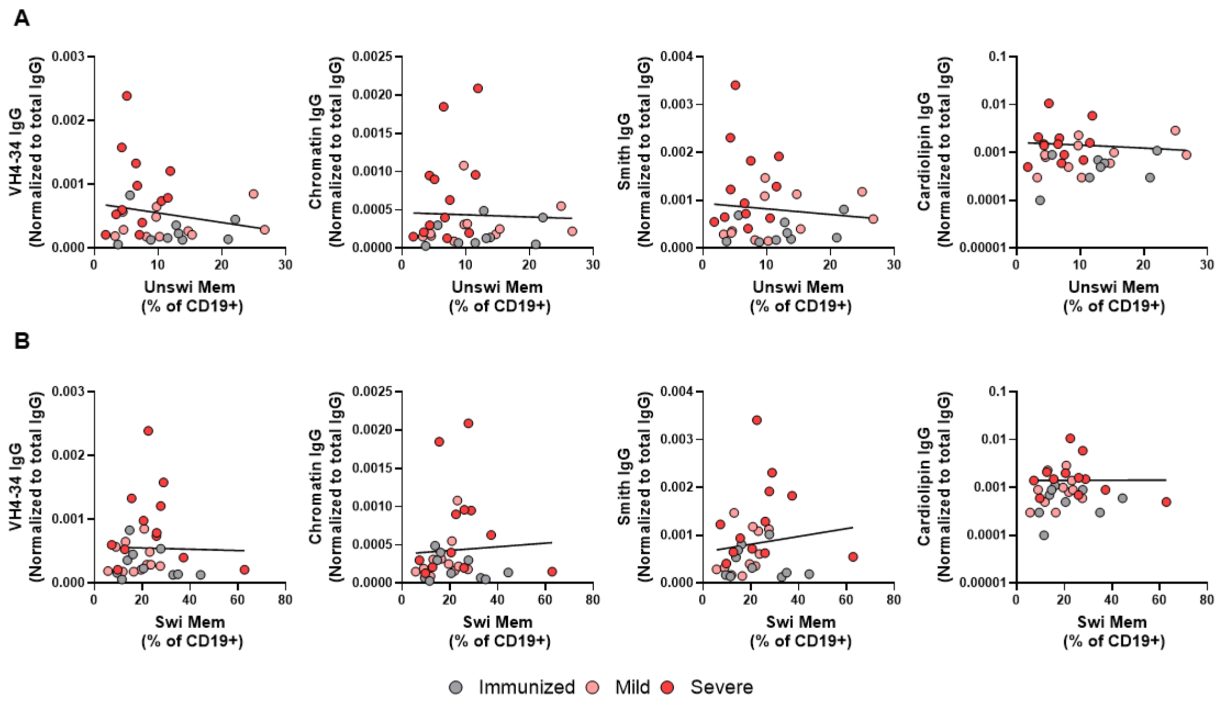

Supplemental Figure 2. Frequency of unswitched or switched memory B cells do not correlate with levels of autoreactive IgG antibodies. (A) Correlation of the frequency of unswitched memory or (B) switched memory B cells with plasma levels of autoreactive IgG antibodies (anti-VH4-34, anti-Chromatin, anti-Smith or anti-Cardiolipin) normalized to total IgG. Statistics: Pearson correlation.

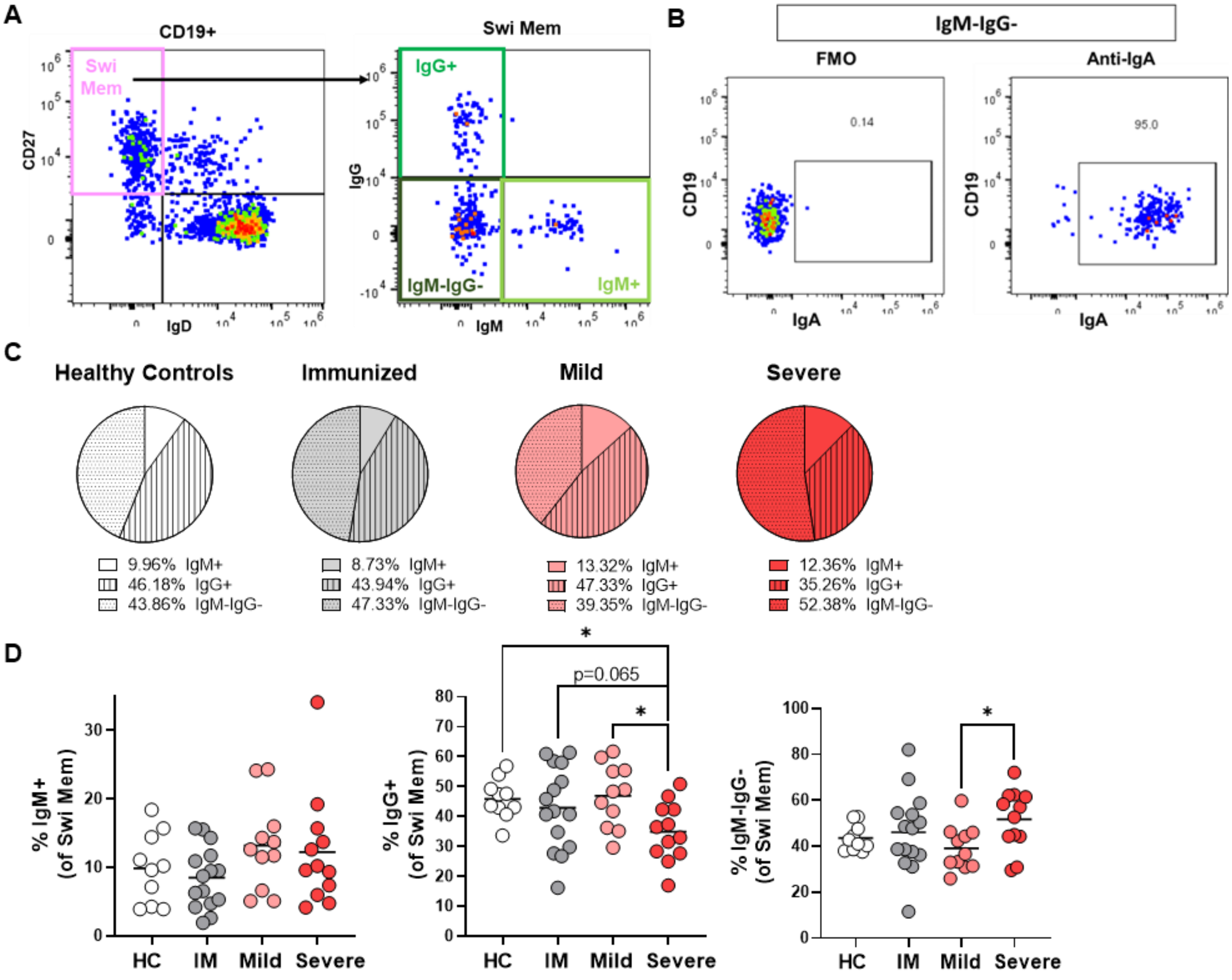

Supplemental Figure 3. Alteration in isotype class of Switched Memory B cells with Severe SARS-CoV-2 infection. (A) Representative flow plot from severe SARS-CoV-2 infection depicting IgM vs IgG gating in switched memory B cells. (B) Representative flow plot from healthy control gated on IgM-IgG- in switched memory B cells showing the majority of this population is IgA+ compared to FMO. (C) Comparison of the average frequency of IgM+, IgG+ or IgM-IgG- cells out of the switched memory compartment (CD27+IgD-) within each cohort. Sample size: Healthy controls (HC; N=10), Immunized controls (IM; N=15), subjects with Mild (N=11) or Severe SARS-CoV-2 infection (N=14). (D) Frequency of IgM+, IgG+ or IgM-IgG- cells within the switched memory population (CD27+ IgD-). Statistics: One-way ANOVA, *$p<0.05$.

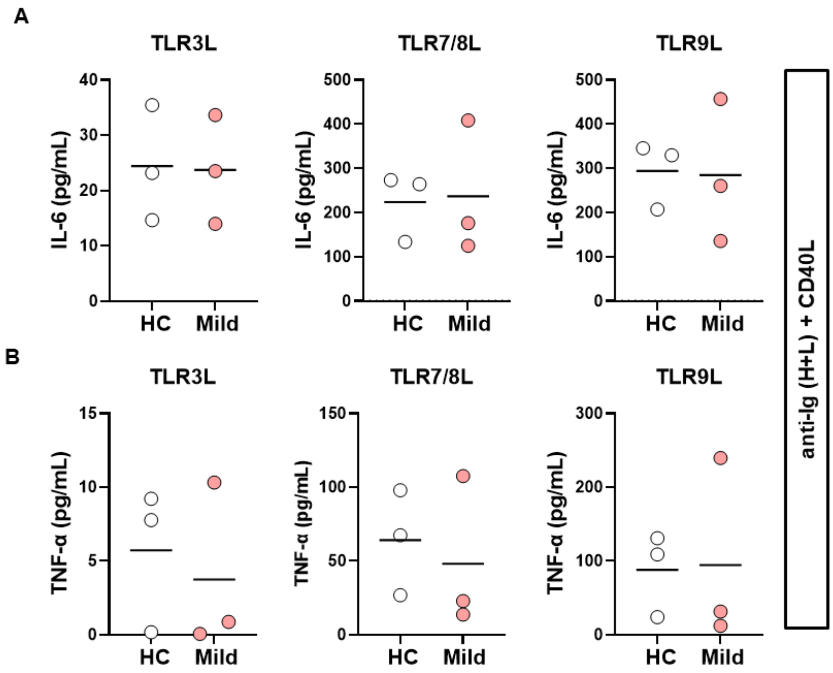

Supplemental Figure 4. No difference in levels of IL-6 and TNFα secretion between B cells from healthy controls or mild SARS-CoV-2 infection. (A) B cells were purified from healthy controls (HC, N=3) or subjects with mild SARS-CoV-2 infection (N=3) then stimulated in vitro overnight with 10ug/mL anti-Ig (H+L) F(ab)’2 + 100ng/mL CD40L with either 1ug/mL TL3RL (Poly I:C), 1ug/mL TLR7/8L (R848), or 2.5ug/mL TLR9L (CpG) and secretion of IL-6 or (B) TNFα was measured in cell culture supernatant. Statistics: unpaired t test.

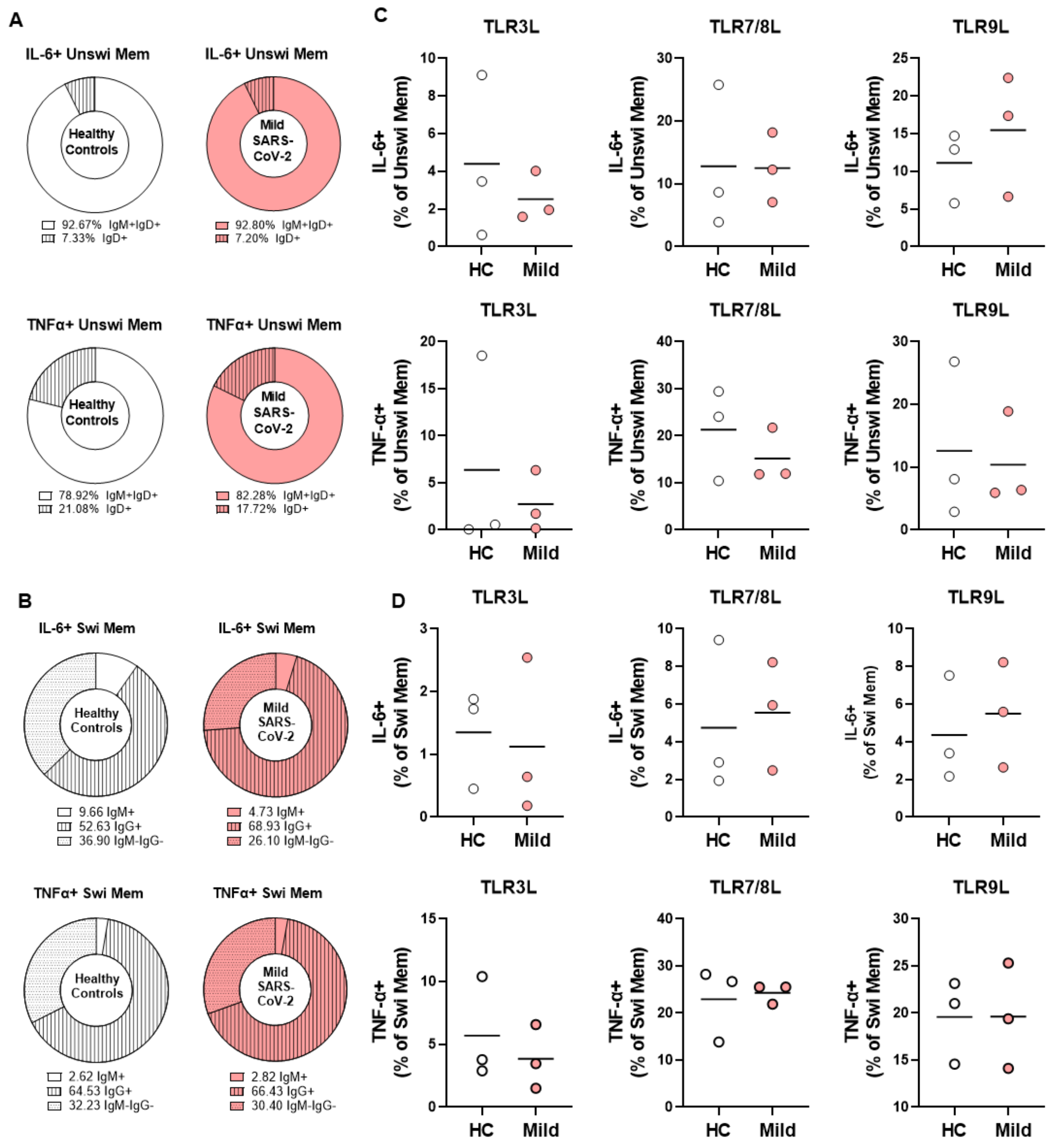

Supplemental Figure 5. Frequency of memory isotype classes and no difference in the frequency of IL-6+ and TNFα+ between memory B cells from healthy controls or mild SARS-CoV-2 infection in response to viral TLR ligands. (A) Average frequency of IgM+IgD+ or IgD-only populations within the IL-6-producing or TNFα-producing unswitched memory B cells or (B) average frequency of IgM+, IgG+, or IgM-IgG- populations within the IL-6-producing or TNFα-producing switched memory B cells after PBMCs from healthy controls (N=3) or subjects with mild SARS-CoV-2 infection (N=3) were stimulated with 50ng/mL PMA+ 1ug/mL Ionomycin or unstimulated control (-) for 4 hours in vitro. (C) PBMCs were stimulated 4hr in vitro with 10ug/mL anti-Ig (H+L) F(ab)'2 + 100ng/mL CD40L with either 1ug/mL TL3RL (Poly I:C), 1ug/mL TLR7/8L (R848), or 2.5ug/mL TLR9L (CpG) and frequency of IL-6+ or TNFα+ unswitched or (D) switched memory B cells from healthy controls (HC, N=3) or subjects with mild SARS-CoV-2 infection (N=3) were enumerated. Statistics: unpaired t test.
